# Surveillance of malaria vector population density and biting behaviour in western Kenya

**DOI:** 10.1186/s12936-015-0763-7

**Published:** 2015-06-17

**Authors:** Ednah N Ototo, Jenard P Mbugi, Christine L Wanjala, Guofa Zhou, Andrew K Githeko, Guiyun Yan

**Affiliations:** Centre for Global Health Research, Kenya Medical Research Institute (KEMRI), PO Box 1578, Kisumu, 40100 Kenya; Kenyatta University, PO Box 43844, Nairobi, Kenya; Masinde Muliro University of Science and Technology, PO Box190-50100, Kakamega, Kenya; Program in Public Health, University of California, Irvine, CA 92697 USA

**Keywords:** Malaria, Vector surveillance, Biting behaviour

## Abstract

**Background:**

Malaria is a great public health burden and Africa suffers the largest share of malaria-attributed deaths. Despite control efforts targeting indoor malaria transmission, such as insecticide-treated bed nets (ITNs) and deployment of indoor residual spraying, transmission of the parasite in western Kenya is still maintained. This study was carried out to determine the impact of ITNs on indoor vector densities and biting behaviour in western Kenya.

**Methods:**

Indoor collection of adult mosquitoes was done monthly in six study sites in western Kenya using pyrethrum spray collections from 2012 to 2014. The rotator trap collections were done in July–August in 2013 and May–June in 2014. Mosquitoes were collected every 2 h between 18.00 and 08.00 h. Human behaviour study was conducted via questionnaire surveys. Species within *Anopheles gambiae* complex was differentiated by PCR and sporozoite infectivity was determined by ELISA. Species distribution was determined and bed net coverage in the study sites was recorded.

**Results:**

During the study a total of 5,469 mosquito vectors were collected from both PSC and Rotator traps comprising 3,181 (58.2%) *Anopheles gambiae* and 2,288 (41.8%) *Anopheles funestus*. Compared to all the study sites, Rae had the highest density of *An. gambiae* with a mean of 1.2 (P < 0.001) while Kombewa had the highest density of *An. funestus* with a mean of 1.08 (P < 0.001). Marani had the lowest density of vectors with 0.06 *An. gambiae* and 0.17 *An. funestus* (P < 0.001). Among the 700 PCR confirmed *An. gambiaes.l.* individuals, *An. gambiaes.s.* accounted for 49% and *An. arabiensis* 51%. Over 50% of the study population stayed outdoors between 18.00 and 20.00 and 06.00 and 08.00 which was the time when highest densities of blood fed vectors were collected. *Anopheles gambies.s.* was the main malaria parasite vector in the highland sites and *An. arabiensis* in the lowland sites. Bed net ownership in 2012 averaged 87% across the study sites.

**Conclusions:**

This study suggests that mass distribution of ITNs has had a significant impact on vector densities, species distribution and sporozoite rate. However, shift of biting time poses significant threats to the current malaria vector control strategies which heavily rely on indoor controls.

## Background

Historically, malaria in the western Kenya highlands has existed. Since the late 1980s, epidemic to hyperendemic malaria has evolved in the western Kenya highlands because of severe public health problems associated with high morbidity and mortality [1–3]. Prior to the 1990s, malaria was managed by chemotherapy. However, following the resistance of *Plasmodium falciparum* to chloroquine and sulfadoxine-pyrimethamine, the country shifted to the use of artemisinin-based combination therapy. Insecticide-treated bed nets (ITNs) and other vector control strategies gained favour based on large-scale randomized control trials. Initial trials with ITNs indicated promising protection and a reduction in morbidity and mortality [4]. However, the affected populations could not afford the ITNs in early 2000. The Kenya Government policy on subsidized ITNs and targeting vulnerable populations increased the number of people who had ITNs in their households but the overall effect on malaria transmission was low [5]. By 2011, the government rolled out the universal bed net programme where every two persons in a household were provided with a free ITN [5]. It was expected that ownership and usage of 80% of ITNs would have a high epidemiological impact on malaria transmission.

This programme has faced numerous challenges, among them insecticide resistance, non-compliant human behaviour, changes in biting habits of the vector, changes in species composition, and vector density. It has been shown that *Anopheles gambiae* has developed resistance to pyrethroids in western Kenya [6]. Biting behaviour has seen a small but significant increase in early biting of malaria vectors in the western Kenya lowlands [7]. The proportion of *Anopheles arabienis* has progressively increased in the western Kenya highlands [8]. Although high ownership of ITNs has been reported in western Kenya, the usage has not been as high [9, 10]. Consequently, this has led to high transmission of the malaria parasite in the population with low usage.

This study was carried out to determine the impact of ITNs on indoor vector densities and biting behaviour in western Kenya. The information from this study can help to inform future malaria control planning.

## Methods

### Study area

**Figure 1:**
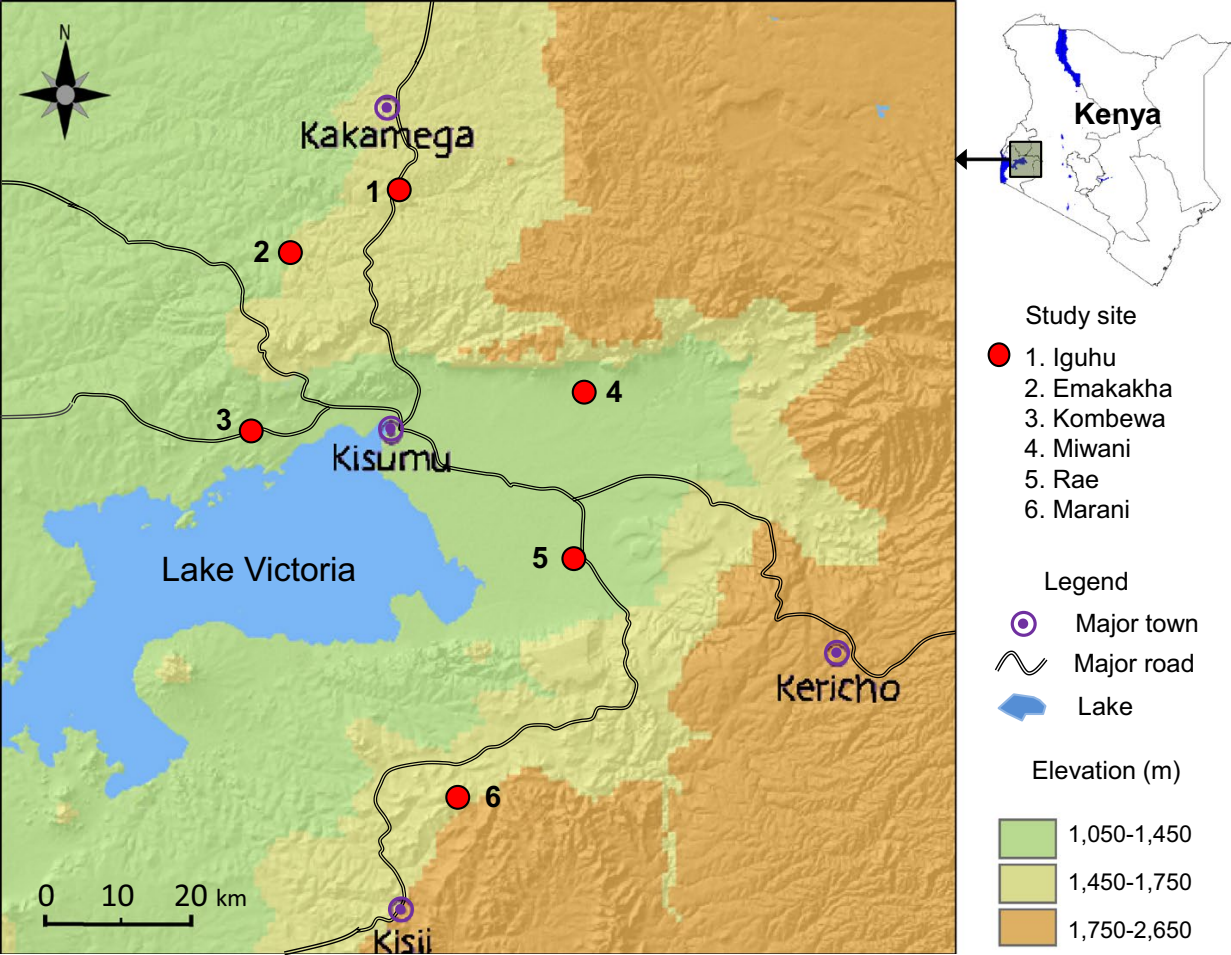
Map showing the study sites in the western Kenya highlands.

The study was conducted in six sites in western Kenya: three highland sites, Iguhu (34°45′E, 0°10′N, 1,430–1,580 m above sea level) which is meso-endemic in Kakamega County; Emakakha (34°64′E, 0°22′N, 1,463–1,604 m asl) in Vihiga County; and, Marani (34°48′E, 0°35′S, 1,520–1,700 m asl) in Kisii County. Three holo-endemic sites were located in lowlands: Kombewa (34°30′E, 0°07′N, 1,150–1,300 m asl); Miwani (0°07′S, 35°05′E, 1,100–1,200 m asl); and Rae (00°25′S 34°95′E, 1,143–1,200 m asl) in Kisumu County (Figure 1). Iguhu and Emakakha have flat shaped valleys, Kombewa, Rae and Miwani are in the flat lowland regions, while Marani has steep valleys [11]. Climate in western Kenya consists mainly of two seasons of rainfall, a long rainy season that is the peak of malaria transmission between March and May and a short one between October and November.

### Adult vector entomological surveys

Pyrethrum spray collections [12] were conducted monthly in 30 randomly selected sentinel houses in each village from January 2012 to June 2014. Mapping of the location of the houses around the study region was done by the global positioning system (GPS) and the geographical coordinates recorded. The number of people sleeping in each house was recorded during mosquito sampling. Mosquitoes collected were morphologically identified as *An. gambiaes.l.* or *Anopheles funestus*. Legs and wings of the female *An. gambiaes.l.* were frozen at −20°C in labelled vials before molecular identification by PCR into *An. gambiaes.s.* or *An. arabiensis* according to Scott et al. [13]. The head and thorax of the mosquitoes were separated from the abdomen and sporozoite ELISA was used to determine their infectivity with *Plasmodium* parasites [14].

### Mosquito activity and biting behaviour

To determine the mosquito active peak hours during night, rotator traps (John W Hock Co, Gainesville, FL, USA) were set up during the dry season in July–August in 2013 and in the wet season in May–June in 2014. These traps were set both indoors and outdoors in three selected sentinel houses. Both indoor and outdoor Traps were set at 1.5 m from the ground. The indoor traps were set at the foot of the bed while the outdoor traps were set at 2 m from the houses.

The rotator traps collected mosquitoes every 2 h from 18.00 to 08.00. Collections were repeated for 5 days in each of the houses in all the study sites. PCR and ELISA were conducted on the collected vectors for the identification of species and sporozoite infectivity.

### Human behavioural study

In the six selected study sites, the human behaviour study was done in 2013 to determine if there is any association between the peak of human outdoor activities and peak of mosquito blood feeding time. A questionnaire survey was administered in 200 randomly selected households where consent of participation had been obtained, during the wet and dry season. The questions asked included the time they went indoors in the evening, time they were outdoors in the morning, the activities that kept them outdoors and if they slept indoors or outdoors.

### Household bed net surveys

Bed net ownership and usage were surveyed accompanying the pyrethrum spray collections. The number of children that slept under nets at night was recorded, including their ages. The type of net (whether treated, untreated or long-lasting nets) was recorded. The condition of the net was recorded to determine whether the net had holes or not.

### Scientific and ethical clearance

This study began after obtaining ethical clearance from the Ethical Review Board at the Kenya Medical Research Institute and University of California, Irvine (SSC no 1382). The area chief, sub-chief and village elders were sensitized on the study activities planned, household heads provided written consent authorizing the spraying of their houses for mosquito collection. Those who were not willing for their houses to be sprayed were excluded from the study. Those who declined participation during the follow-up time were dropped from the study and new participants recruited.

### Statistical analysis

Monthly adult anopheline mosquito abundance was calculated as the number of female mosquitoes per house per night. Bed net ownership rate was calculated as the ratio of the number of households with at least one bed net over the total number of households surveyed. The average abundance of anopheline mosquitoes in a house was computed from January 2012 to August 2014 for each site. Overall differences in mosquito densities among villages were compared using Kruskal–Wallis ANOVA (by ranks) and median test. Between-village difference in mosquito densities was compared using multiple comparisons of mean ranks for all groups. Analysis was done using STATISTICA 10 (Dell STATISTICA, Austin, TX, USA).

## Results

### Vector population dynamics

**Figure 2:**
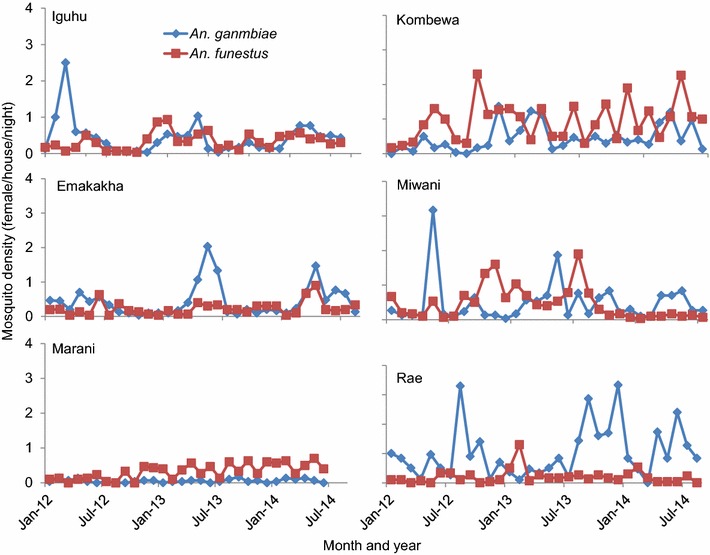
*Anopheles gambiae* and *Anopheles funestus* indoor resting densities in the different study sites.

In the study duration, between January 2012 and August 2014, a total of 5,469 mosquito vectors were collected from both PSC and rotator traps; 3,181 (58.2%) were *An. gambiae* and 2,288 (41.8%) were *An. funestus*. In the highland sites, *An. gambiae* was the most abundant vector while in the lowland sites *An. arabiensis* and *An. funestus* were the most abundant vectors collected. Densities of *An. gambiae* peaked in April to May in the study sites (Figure 2). These peaks were reached generally 1 month after the onset of the long rainy season, which is the main malaria transmission season. Generally, low vector densities were seen during the dry season between January and March, however Rae showed different patterns of vector abundance, where *An. gambiae* was high throughout the study period (Figure 2). *Anopheles funestus* peaked 2 months after the rainy season between June and July (Figure 2).

The lowland site of Rae had the highest mean *An. gambiae* indoor resting densities of 1.02, Marani in the highlands had the least at 0.06 mean vectors. There was no difference in vector abundance in the other four study sites as shown in Table 1. *Anopheles funestus* indoor resting densities were highest in Kombewa (1.08). Miwani had fewer *An. funestus* vectors than Kombewa (0.48) but higher than the other four sites, which had similar means when compared. Marani had the least resting densities of 0.17 as shown in Table 1.

**Table 1:** Mosquito densities by village and mosquito species

Site	Density (females/trap/night) (95% CI)	
	*An. gambiae*	*An. funestus*
Iguhu	0.47 (0.41, 0.52)	0.25 (0.21, 0.29)
Emakakha	0.44 (0.37, 0.51)	0.23 (0.18, 0.28)
Kombewa	0.65 (0.57, 0.73)	1.08 (0.96, 1.21)
Miwani	0.48 (0.38, 0.58)	0.48 (0.39, 0.56)
Rae	1.02 (0.93, 1.12)	0.20 (0.16, 0.23)
Marani	0.06 (0.04, 0.07)	0.17 (0.11, 0.23)

*Anopheles gambiae* density in Rae was significantly higher (P < 0.001) than anywhere else. Densities in Kombewa, Emakakha, Iguhu and Miwani were not significantly different from each other. Density in Marani was the lowest. Kombewa had the highest density of *An. funestus* (P = 1.000) that was significantly higher than any other place. Densities in all other places were not significantly different as shown in Tables 1 and 2.

**Table 2:** Significance (2-tailed p values) of multiple comparisons of mean ranks for all groups

	Iguhu	Emakakha	Kombewa	Miwani	Rae	Marani
Iguhu		1.000	1.000	1.000	*<0.001*	*<0.001*
Emakakha	1.000		0.155	1.000	*<0.001*	*<0.001*
Kombewa	*<0.001*	*<0.001*		*0.047*	*<0.001*	*<0.001*
Miwani	*0.006*	*0.011*	*<0.001*		*<0.001*	*<0.001*
Rae	1.000	1.000	*<0.001*	*0.007*		*<0.001*
Marani	1.000	1.000	*<0.001*	0.259	1.000	

Above diagonal: *An. gambiae*; below diagonal: *An. funestus*.

### Rotator trap collections

**Figure 3:**
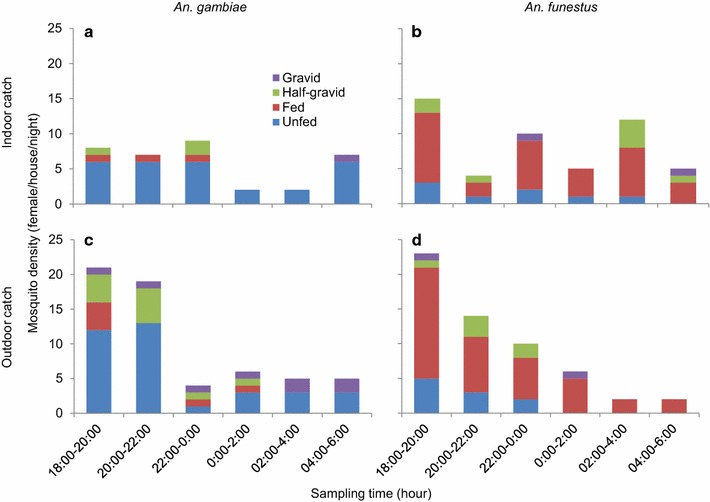
Comparisons between indoor and outdoor vector densities.

*Anopheles gambiae* and *An. funestus* were the main vectors collected in the outdoor rotator traps; 58% of the *An. gambiae* vectors collected were not fed, 10% were freshly fed, 18% were half gravids while 14% were gravids; 17% of *An. funestus* were not fed, 68% were freshly fed, 11% were half gravids and 4% were gravids; 45% of the vectors collected between 18.00 and 20.00 h were blood fed and 39% were unfed. Before dawn, 43% of the vectors collected were not fed; 28% of the vectors collected were fed and gravid, respectively. This shows that vectors feed in the early hours before dawn or after dusk. 68% of *An. funestus* collected were mostly blood fed. This species feeds at all times both indoors and outdoors, as shown in Figure 3. The gravid vectors collected, as shown in Figure 3, were either coming out to look for resting habitats or they were unfed (Figure 3). 80% of the *An. gambiae* collected from the indoor traps were unfed, 9% were fed, 8% half gravid and 3% were gravids. 16% of the *An. funestus* vectors collected were unfed, 64% were fed, 16% were half gravid and 4% were gravid.

### Human behaviour study

**Figure 4:**
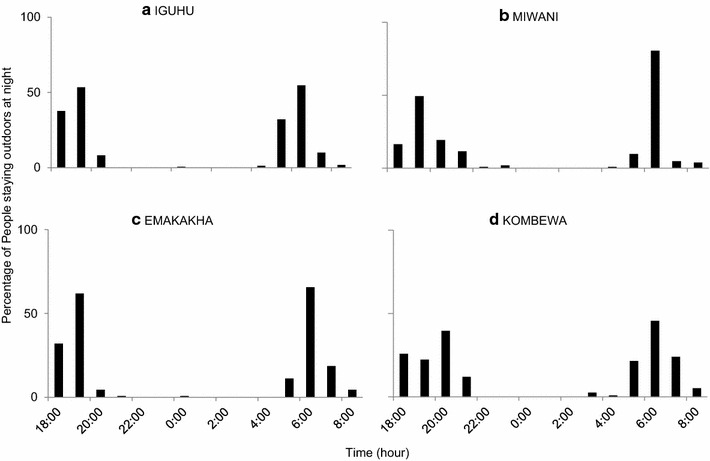
Human behaviour study.

The data collected from the questionnaires administered in the households’ show that in the highland sites, 40% of the population stayed out between 18.00 and 20.00 and 50% of the population was outdoors in the dawn hours between 04.00 and 06.00. This is because the population practices agriculture and they woke up early to go to the farms. In the lowland sites, 45% of the population was out in the evening (18.00–20.00) while 50% of the population woke up after 06.00. The results show that no one slept outdoors, even in the hot months, showing a low risk of outdoor malaria transmission (Figure 4).

### Variation in vector species

**Figure 5:**
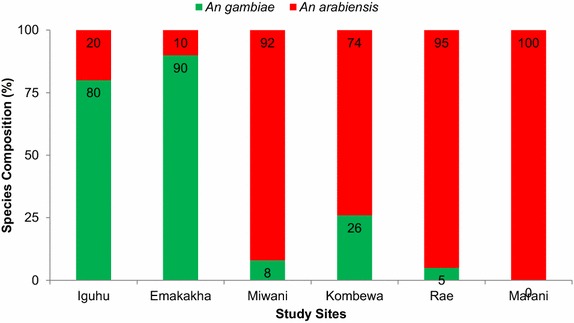
Species composition the study sites.

Out of the vectors collected, 700 *An. gambiaes.l.* were successfully tested by PCR. *Anopheles gambiaes.s.* was the predominant species in the highlands (90%) while in the lowland sites *An. funestus* and *An. arabiensis* were the most dominant species. Miwani had 8% *An. gambiae* while Marani had 100% *An. arabiensis* (Figure 5) and a mean indoor resting density of 0.17 *An. funestus. Anopheles funestus* has emerged as the main vector of transmission in Marani as it was the main vector collected.

### Vector infectivity

In the highland sites, *An. gambiae* is the main vector of transmission with the highest sporozoite rate of 1.6 in Emakakha. In the lowland sites, *An. funestus* is the main vector of transmission with the highest sporozoite rates of 2.4. Emakakha had the most infectious vectors with *An. funestus* sporozoite rates as high as 5.3 (Table 3).

**Table 3:** Vector infectivity in the study sites

Site	Iguhu	Marani	Emakakha	Kombewa	Rae	Miwani
Total mosquitoes analysed	274	65	267	220	180	163
*An. gambiae* (sporozoite +ve)	251 (2)	25 (1)	248 (4)	136 (1)	175 (1)	147 (1)
Sporozoite rate	0.8	4	1.6	0.7	0.6	0.7
*An. funestus* (sporozoite +ve)	23 (0)	40 (0)	19 (1)	84 (2)	5 (0)	16 (0)
Sporozoite rate	0	0	5.3	2.4	0	0

### Bed net ownership

**Figure 6:**
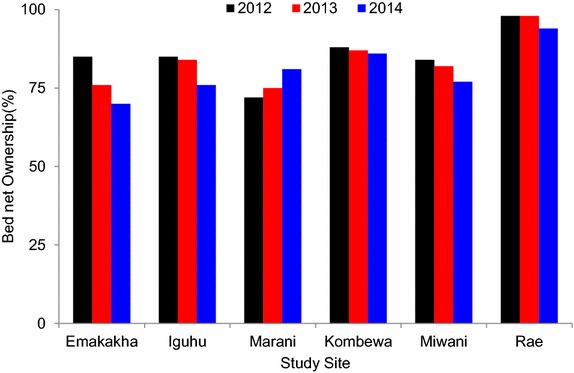
Bed net ownership in the study sites.

**Figure 7:**
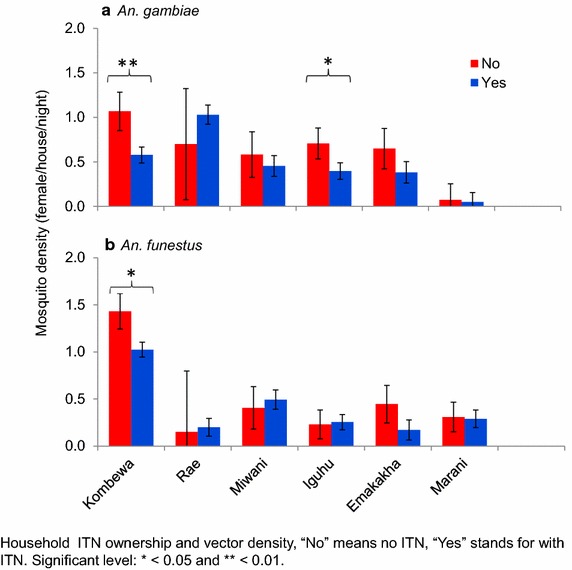
Analysis of vector densities between households with bed nets and those without bed nets.

There was a high bed net ownership of up to 80% in the study areas, both in the highland and lowland regions. The lowland site Rae reported the highest ownership of 98% (Figure 6). There was a significant difference in the number of vectors collected in Kombewa and Iguhu between households that owned at least one bed net and those that did not (Figure 7). This could be as a result of the community effect as a result of the high bed net coverage.

## Discussion

This study was carried out to assess the impact of ITNs on indoor vector densities and biting behaviour in western Kenya as the use of ITNs has been shown to be effective in reducing mortality and malaria transmission in the past [15]. Before the mass distribution of ITNs in 2011, bed net ownership in western Kenya was reported to be below 80% and parasite resurgence had been seen in areas of western Kenya. This was attributed to vector resistance to pyrethroids and the inefficacy of bed nets because of the low ownership [16]. Afterwards, the Roll Back Malaria Partnership raised coverage of ITNs to ≥80% through the free mass distribution of long-lasting insecticidal nets (LLINs)/ITN campaigns, which were carried out in various parts of Africa [5]. The current policy for vector control in Kenya includes the use of LLINs and limited use of IRS where the government-marketed, subsidized bed nets in 2002, 2006 to vulnerable groups until 2011 when there was a universal distribution policy was implemented with every two persons in a household receiving a free bed net [17].

In this study, high bed net ownership of >80% in all six sites was confirmed. The high bed net ownership has a community effect where people without nets are protected by the area-wide effects of ITNs nearby [18]. *Anopheles gambiae* indoor resting densities over the last decade have decreased tenfold in Iguhu, sixfold in Marani and fourfold in Kombewa, while densities of *An. funestus* have decreased threefold in Iguhu and sixfold in Kombewa. However in Marani, over the decade densities of *An. funestus* have increased threefold compared to a study by Ndenga et al. [19]. Likewise, sporozoite rates of *An. gambiae* have declined fourfold in Iguhu and Kombewa while they have increased fourfold in Marani. *Anopheles funestus* sporozite rates remained constant in Kombewa, while in the other sites there were no confirmed infectious *An. funestus*.

*Anopheles funestus* is seen as one of the most abundant vectors in Kombewa, which has been reported previously [16]. The species is re-emerging in Marani where it was the most abundant species, as shown in the results. *Anopheles funestus* breeds in permanent habitats towards the end of the wet season and is known to require vegetation and shade and the larval habitats are found mainly in swamps and pastures [20]. *Anopheles funestus* takes 3 weeks to mature, which is longer than *An. gambiae* maturation period. Other studies conducted in western Kenya lowland region have reported that *An. funestus* is re-emerging, which is suspected to be as a result of pyrethroid resistance after a long-term implementation of ITNs [21]. Previous studies in the sugar-belt region of Miwani reported the ratio of *An. arabiensis* to be higher than that of *An. gambiaes.s*., especially during the dry season [22]. The use of ITNs has had a great impact on densities, species and sporozoite rates. The proportion of *An. arabiensis* is increasing in the highlands [23], a factor that could have malaria transmission implications as *An. arabiensis* is a less efficient vector than *An. gambiae,* as *An. arabiensis* is zoophilic [24].

Githeko et al. [25] found that malaria vectors fed during the late part of the night with peaks at 05.00 h. In this study, *An. gambiae* caught after midnight was blood fed, while fed *An. funestus* were caught throughout the night both indoors and outdoors. It is likely that blood-fed *An. funestus* may have been avoiding resting. This observation supports exophilic behaviour in *An. funestus,* a phenomenon that requires further investigation. In regard to human and mosquito activity, data from this study suggests that there is a risk of transmission at dusk and at dawn. Data collected during the study did not support continuous outdoor transmission since the majority of humans were indoors between 21.00 and 05.00 h.

The use of LLINs has been reported to change the feeding and resting behaviour of mosquitoes [7]. The study reports similar findings that there was high host seeking activity of the vectors at around 18.00 and 20.00 that led to earlier feeding in *An. gambiae* populations. This could be as a result of the use of ITNs. *Anopheles funestus* showed no change in feeding habits as the results show that they bite throughout the night both indoors and outdoors. This poses a great risk of malaria transmission throughout the night despite high bed net coverage. Studies done by Oloo et al. [26] showed that the use of permethrin-treated sisal curtains led to the exit of half-gravid mosquitoes from indoors. This could be one of the reasons why there was a high collection of half-gravid mosquitoes, both *An. gambiae* and *An. funestus*. This result coincided with the human behaviour study where >50% of the population stayed outdoors after dusk but went indoors by 21.00 h and woke up before dawn to do their daily chores. Similar studies in the lowlands of western Kenya have also reported that the vectors bite throughout the night and mostly indoors [27]. *Anopheles funestus* feeding habits suggest that transmission is most likely happening indoors, although there is a high risk of outdoor transmission. Findings in the lowland regions show that *An. funestus* was the most infectious vector while in the highlands; *An. gambiae* was the main vector of transmission. The ratio of blood-feds to half-gravids was 3:1 and the capture of both blood-feds and half-gravids shows that there was insecticidal excito-repellency. This blood-fed to gravid ratio can be as a result of mortality of the vectors after contact with ITNs or insecticide repellency. The capture of half-gravids and gravid vectors is an indicator of exophily.

Vector biting and resting behaviour may be altered by exposure to insecticides. Under the use of ITNs in Tanzania, the tendency of mosquitoes to exit the indoor environment increased [28]. In Ethiopia, *An. arabiensis* avoided resting on DDT sprayed surfaces [29]. In western Kenya, the proportion of *An. gambiae* taking a blood meal before humans slept under ITNs increased after the introduction of ITNs [7]. These shifts in biting and resting behaviour reduce exposure of malaria vectors to the impacts of insecticides thus minimizes their mortality resulting in sustained malaria transmission. Data collected from this study suggests *Anopheles funestus* may have changed its resting behaviour. In previous studies, where ITNs were not in use [22], no blood fed females of *An. funestus* were collected in light traps and exit traps. Equally, no blood fed female *An. funestus* were collected in outdoor placed light traps. In the current study, blood fed *An. funestus* were collected in indoor and outdoor placed light traps suggesting a post blood feeding flight activity and possibly exit to the outdoor environment. Studies being undertaken will test whether the blood fed females had fed on humans or other hosts. Data from this study indicates that the proportion of *An. funestus* in Iguhu and Marani, where historical data exists, has increased in recent years which suggests that this vector has better survival under the use of ITNS than *An. gambiae s.l.* this could be explained by increased avoidance of insecticide treated surfaces a behaviour that remains to be studied.

There was a difference in the densities of the vectors collected between households that had at least one bed net and households that did not own a bed net. This was seen in Kombewa, a site that had *An. funestus* as the main vector. Fewer vectors were collected in the households that had at least one bed net. Mbogo et al. [30] reported that after distribution of permethrin-treated bed nets, fewer vectors were collected. This shows that owning a bed net protects the household from malaria vectors, while a high coverage of bed net ownership creates community-wide protection from mosquitoes. Besides owning a bed net and the high distribution in the study sites, there has been a reported increase in the resistance to insecticides both in West Africa [31] and in East Africa, especially in western Kenya [6]. Change in behaviour patterns due to high ownership of bed nets [7] are also reducing the role of *An. gambiaes.s.* in malaria transmission but not ruling out the role of *An. arabiensis* [32].

## Conclusions

High bed net coverage has been observed in the highlands and lowland sites and this may explain the decreasing indoor resting densities and sporozoite rates. ITN pressure may be greater on *An. gambiae* compared to *An. arabiensis* as *An. arabiensis* is more zoophilic and exophilic. The relatively high indoor resting densities of *An. funestus,* despite high ITN coverage, is puzzling as the susceptibility of *An. funestus* to insecticides in these sites remains unknown. This phenomenon requires further research. The high abundance of the blood-fed fraction of *An. funestus* in the rotator traps, both indoors and outdoors suggests exophillic behaviour which in turn shows that there is an increased survival of *An. funestus*, a highly efficient vector which is a threat to malaria control. This study suggests that mass distribution of ITNs has had a significant impact on transmission; however active surveillance on vector dynamics is required in order to identify emerging risks.
